# Genetic diversity and population structure of *Plasmodium falciparum* in Nigeria: insights from microsatellite loci analysis

**DOI:** 10.1186/s12936-021-03734-x

**Published:** 2021-05-26

**Authors:** Fehintola V. Ajogbasile, Adeyemi T. Kayode, Paul E. Oluniyi, Kazeem O. Akano, Jessica N. Uwanibe, Benjamin B. Adegboyega, Courage Philip, Oluwagboadurami G. John, Philomena J. Eromon, George Emechebe, Finimo Finimo, Nnenna Ogbulafor, Nma Jiya, Uche Okafor, Jose Ambe, Robinson D. Wammanda, Stephen Oguche, Olugbenga A. Mokuolu, Akintunde Sowunmi, Onikepe A. Folarin, Christian T. Happi

**Affiliations:** 1 African Centre of Excellence for Genomics of Infectious Diseases (ACEGID), Redeemer’s University, Ede, Nigeria; 2 Department of Biological Sciences, Faculty of Natural Sciences, Redeemer’s University, Ede, Nigeria; 3 Department of Biological Sciences, Covenant University, Ota, Nigeria; 4 Department of Paediatrics, Imo State University Teaching Hospital, Orlu, Nigeria; 5 Department of Paediatrics, Federal Medical Centre, Yenagoa, Nigeria; 6 Case Management Unit, National Malaria Elimination Programme, Federal Ministry of Health, Abuja, Nigeria; 7 Department of Paediatrics, Uthman Dan Fodio University, Sokoto, Nigeria; 8 Department of Paediatrics, University of Nigeria Teaching Hospital, University of Nigeria, Nsukka, Nigeria; 9 Department of Paediatrics, University of Maiduguri, Maiduguri, Nigeria; 10 Department of Paediatrics, Ahmadu Bello University, Zaria, Nigeria; 11 Department of Paediatrics, University of Jos Teaching Hospital, University of Jos, Jos, Nigeria; 12 Department of Paediatrics and Child Health, University of Ilorin, Ilorin, Nigeria; 13 Institute of Medical Research and Training, College of Medicine, University of Ibadan, Ibadan, Nigeria; 14 Department of Pharmacology and Therapeutics, College of Medicine, University of Ibadan, Ibadan, Nigeria

**Keywords:** Malaria, *Plasmodium falciparum*, Genetic diversity, Microsatellite, Nigeria

## Abstract

**Background:**

Malaria remains a public health burden especially in Nigeria. To develop new malaria control and elimination strategies or refine existing ones, understanding parasite population diversity and transmission patterns is crucial.

**Methods:**

In this study, characterization of the parasite diversity and structure of *Plasmodium falciparum* isolates from 633 dried blood spot samples in Nigeria was carried out using 12 microsatellite loci of *P. falciparum*. These microsatellite loci were amplified via semi-nested polymerase chain reaction (PCR) and fragments were analysed using population genetic tools.

**Results:**

Estimates of parasite genetic diversity, such as mean number of different alleles (13.52), effective alleles (7.13), allelic richness (11.15) and expected heterozygosity (0.804), were high. Overall linkage disequilibrium was weak (0.006, P < 0.001). Parasite population structure was low (Fst: 0.008–0.105, AMOVA: 0.039).

**Conclusion:**

The high level of parasite genetic diversity and low population structuring in this study suggests that parasite populations circulating in Nigeria are homogenous. However, higher resolution methods, such as the 24 SNP barcode and whole genome sequencing, may capture more specific parasite genetic signatures circulating in the country. The results obtained can be used as a baseline for parasite genetic diversity and structure, aiding in the formulation of appropriate therapeutic and control strategies in Nigeria.

**Supplementary Information:**

The online version contains supplementary material available at 10.1186/s12936-021-03734-x.

## Background

Although the incidence of malaria infections and malaria-associated mortality has reduced in many African countries [1–3], transmission continues in endemic regions despite intensified efforts towards prevention, control and eradication [4, 5]. This is due, in part, to the high genetic diversity of *Plasmodium falciparum* that contributes to increased transmission rate and spread of resistant parasites [6]. Therefore, understanding the extent of genetic diversity, transmission intensity, and parasite population structure in Nigeria—the most malaria burdened country—is essential if the goal of malaria control or elimination is to be achieved.

Molecular techniques play important roles in the analyses of genetic diversity, transmission dynamics, and population structure of *P. falciparum* field isolates. Early molecular studies focused mostly on the use of polymorphic markers, such as merozoite surface protein 1 (*msp*-1), merozoite surface protein 2 (*msp*-2) and glutamate-rich protein (*glurp*) to characterize *P. falciparum* genetic diversity and structure in Nigeria [7–9]. These markers were also useful in monitoring drug efficacy with regards to classification of recurrent *P. falciparum* parasitaemia as re-infection or recrudescent infection [6, 10, 11]. However, there have been contrasting reports of polymorphisms in MSP-1 and MSP-2 in earlier studies in Nigeria [6, 12–14], which is associated with the fact that these antigenic markers are often under intense immune pressure [15–17]. The genotyping results provided by these markers can, therefore, potentially lead to a masked and distorted view of the population structure and transmission patterns which may account for observed variations across parasite populations circulating in a given environment [6].

Microsatellite loci have been suggested to be better alternatives to *msp-1, msp-2* and *glurp* due to their abundance, putative neutrality and higher levels of polymorphisms [18]. This molecular technique remains one of the most efficient and reliable methods for analyzing the genetic diversity of falciparum populations for epidemiological and drug efficacy purposes within countries and across continents [19]. In past studies of using microsatellite analyses, it was observed that parasites from areas of low malaria transmission [19] (< 1% infection) have less genetic diversity but more population structure and greater linkage disequilibrium (i.e., more non-random association among alleles across multiple loci) [4, 19–21]. Contrary, in regions of high malaria transmission, individuals are more likely to be infected by more than one *P*. *falciparum* parasite thereby resulting in an increase in the rate of recombination and subsequently, highly diverse population with low linkage disequilibrium [18, 19, 22]. Although, some studies report a deviation from the norm whereby high levels of heterozygosity (a measure of genetic diversity) is observed in several low transmission countries [18, 23, 24]. This suggests that a high level of heterozygosity may reflect past human demographic processes as opposed to recent epidemiological factors [25].

The objective of this study was to investigate the genetic diversity of circulating *P. falciparum* parasites and their population structures in Nigerian children 6–96 months old with uncomplicated infections, treated with artemisinin-based combination therapy (ACT).

## Methods

### Study site

This is a retrospective, cross-sectional, community-based study that is part of a larger drug therapeutic efficacy testing (DTET) study for monitoring anti-malarial efficacy of artesunate-amodiaquine (AA), artemether-lumefantrine (AL) and dihydroartemisinin-piperaquine (DHP) in the treatment of uncomplicated *P. falciparum* infections in children aged 6–96 months old. A cohort of children were enrolled from nine sentinel sites of the National Malaria Elimination Program of the Federal Ministry of Health (located in six geographical zones of Nigeria), namely; Numan (n = 48), Bodinga (n = 50), Kura (n = 100), Barkin Ladi (n = 100), Ilorin (n = 58), Ibadan (n = 50), Otuasegha (n = 45), Agbani (n = 100) and Ogwa (n = 82) in Adamawa, Sokoto, Kano, Plateau, Kwara, Oyo, Bayelsa, Enugu and Imo States, respectively.

### Study population

Children aged 6–96 months old were eligible for enrollment in the efficacy study if they had symptoms compatible with uncomplicated malaria such as fever, anorexia, vomiting or abdominal discomfort with or without diarrhea with *P. falciparum* infections.

### Sample collection

Filter papers containing dried blood spots (DBS) obtained from 633 children confirmed as malaria positive by microscopy were randomly selected for this study. All DBS samples were collected in 2014 (Adamawa, Bayelsa, Imo, Kwara, Oyo and Sokoto) and 2018 (Enugu, Kano and Plateau). Samples were collected for a duration of three months (July–September) which represents intense malaria transmission season in Nigeria. Two to three drops of finger-pricked blood samples were blotted on 3 mm Whatman filter paper (Whatman International Limited, Maidstone, UK) before treatment initiation (Day 0). The blood samples impregnated on to filter papers were allowed to air-dry properly at room temperature, and DBS were kept in airtight envelopes with silica gel at room temperature until analysed.

### DNA extraction

DNA was extracted from DBS for parasite genetic diversity and population structure studies as previously described [26]. DNeasy Blood and Tissue extraction kit (Qiagen, Germany) was used to extract parasite DNA from DBS following the manufacturer's protocol.

### *Plasmodium falciparum* genotyping by microsatellite loci analysis

Semi-nested PCR amplification of 12 *P. falciparum* microsatellite loci was done using a previously described protocol [17]. The 12 microsatellite loci were Poly A, PfG377, TA81, ARA2, TA87, TA40, TA42, 2490, TA1, TA60, TA109 and PfPk2 [27]. FAM, YAK YELLOW, and ATTO550N-labeled PCR products for the different loci amplified were pooled together for electrophoresis on the ABI 3500XL Genetic Analyzer at the African Centre of Excellence for the Genomics of Infectious Diseases (ACEGID), Redeemer’s University Ede, Osun State, Nigeria. Peakscanner (Applied Biosystems) and GeneMarker (Softgenetics) software were used for normalization across runs and automatic determination of allele length and peak heights in samples containing multiple alleles per locus.

### Data analysis

Microsatellite data was retrieved from the Genetic Analyzer 3500XL and formatted in Microsoft excel (version 16.44) as previously described [28]. Subsequent genetic analysis was only done on samples where all microsatellite markers were successfully amplified. Multiple alleles at a given locus was assumed if minor peaks observed were more than 20% the height of the predominant peak [6]. Although minor alleles were scored in samples containing them, only the predominant alleles were considered for all population genetic and structure analysis. Multi-clone infections were defined as those that had at least two loci containing multiple alleles (only samples with two alleles were included for analysis), while single clone infections were defined as those containing one allele for all microsatellite loci or when one locus contained multiple alleles [6]. Haplotypes were computed using ARLEQUIN software version 3.11 [29] from both single-clone and multi-clone infections. The predominant allele at each locus was used to define twelve-locus parasite haplotype in multiple-clone infections [30].

### Measures of parasite genetic diversity

#### Number of effective alleles (Ne) and number of different alleles (Na)

The number of effective alleles (Ne) and number of different alleles (Na) were computed per locus for each State involved in the study using GENALEX 6.5 [28].

#### Allelic richness

Allelic richness (Ar) was computed using FSTAT (v 3.1) as the average number of alleles per locus [31].

#### Expected heterozygosity

The expected heterozygosity (He), a measure of parasite genetic diversity, represents the probability of being infected by two parasites with different alleles at a given locus, was calculated using ARLEQUIN software version 3.11 [29] with the formula:

1 $${\text{He}}\; = \;[\left( {{\text{n}}/{\text{n}}\, - \,{1}} \right) \, ({1}\, - \,\Sigma {\text{p}}^{{2}} )]$$

where n is the number of isolates analysed, and p represents the frequency of each different allele at a locus [29]. The He values range from 0 to 1. Values closer to 0 indicate little or no genetic diversity while values closer to 1 indicate high genetic diversity.

### Measures of parasite population differentiation

#### Analysis of molecular variance (AMOVA)

Inter- and intra-population variance was determined with analysis of molecular variance (AMOVA, i.e., ΦPT). ΦPT value of zero (0) is considered indicative of no genetic differentiation among populations.

#### Fixation index (Fst)

The population divergence was measured by calculating the fixation index (Fst) for all pairs of parasite population in each State using the GENALEX 6.5 software. An Fst value between 0–0.05 was classified as little genetic differentiation, 0.05–0.15: moderate genetic differentiation, 0.15–0.25: great genetic differentiation and values greater than 0.25 represented very great genetic differentiation [32].

#### Cluster analysis

A Bayesian model implemented in the program STRUCTURE v2.3 [33] was used to determine the number of populations or genetic clusters present in Nigerian States considered in this study. A linked model with admixture was used with 5 replicates for each value of k (from 2 to 6), and a burn-in period of 50,000 iterations of Monte Carlo Markov chains [34]. To obtain the optimal number of genetic populations, estimation of ΔK described by Evanno analysis was done using Structure Harvester [35].

#### Linkage disequilibrium (LD)

Multilocus linkage disequilibrium measured as the standardized index of association (I^S^A) was calculated using the program LIAN version 3.5 [36] for the whole dataset and a data- subset with haplotypes from only confirmed single-clone infections, as a precaution against the bias that may result from presence of any false dominant haplotypes [37]. This index was calculated as:

2 $$\left( {I^{S}_{A} } \right) \, = \, ({1}/{\text{n}}{-}{1 }(\left( {{\text{VD}}/\left( {{\text{VE}}} \right){-}{ 1}} \right)$$

where VE is the expected variance of the nth number of loci for which two individuals differ. VD is the observed variance. Randomization test was done to determine whether the ratio of VD/VE was significantly higher than 1.

## Results

### Demographics and baseline characteristics

Overall, 329 (51.97%) were male and the mean age of all children included in the study was 48.4 ± 15.8 months. Also, mean enrollment body temperature was 37.5 ± 2.5 °C. Overall geometric mean asexual parasitaemia was 16,219 μL^−1^ (range: 2003–198,200).

### Parasite genetic diversity

**Fig. 1:**
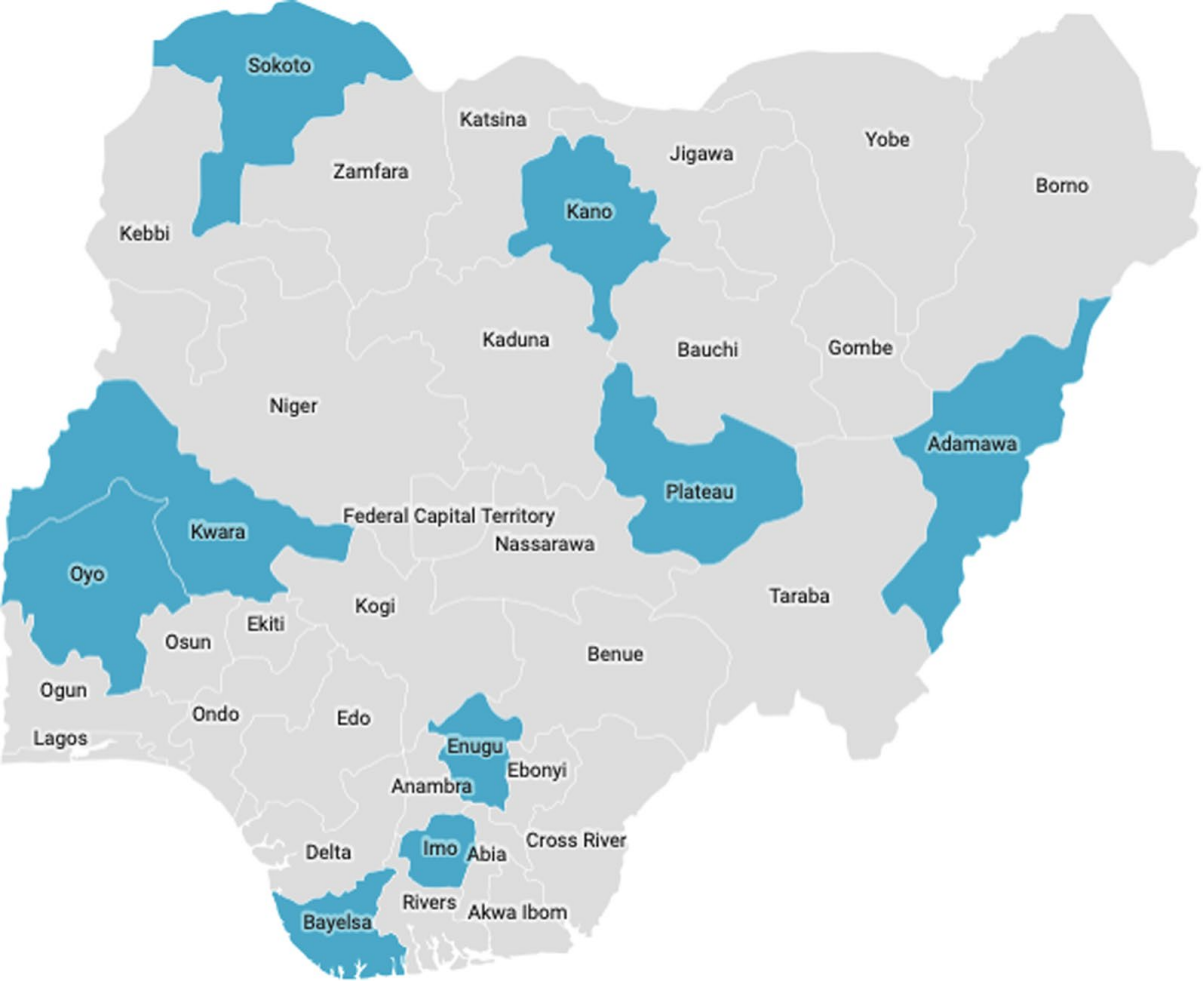
Geographic representation of sites (highlighted) in Nigeria where samples in this study were collected

Of the 633 samples considered for analysis, microsatellite amplification was successful in 571 (90.2%). Most (67.1%) samples were multi-clone infections. There were as many haplotypes as the isolates fully genotyped in the dataset (i.e., all haplotypes were unique). The mean (computed as the average of the sum of values from each locus) of different alleles (Na), effective alleles (Ne), allelic richness (Ar) and expected heterozygosity (He) were 13.52 (+ 0.59), 7.13 (+ 0.42), 11.15 (+ 4.34) and 0.804 (+ 0.013), respectively, when all States were considered as a single population. State-wise, the number of different alleles (Na) (computed as the average of the sum of Na values from each locus) ranged from 11.4 to 17.3. Likewise, the number of effective alleles (Ne) (computed as the average of the sum of Ne values from each locus) ranged from 6.2 to 8.2 (Table 1). The mean allelic richness (Ar) (computed as the average of the sum of Ar values from each locus) ranged from 7.09 to 14.27 (Table 1) and the mean He values observed in each population (computed as the average of the sum of He values from each locus) ranged from 0.776 to 0.842 (Table 1). Although genetic diversity was high in all populations, it was observed that Ar was especially high in parasite populations from two States (Enugu and Kano) obtained in 2018 (Fig. 1).

**Table 1:** Summary of measures for parasite genetic diversity

	AD	BY	EN	OY	IM	KN	KW	SK	PL	Combined
Na	13.2	12.3	16.3	11.4	11.7	17.3	14.4	12.4	12.8	13.52 (+ 0.59)
Ne	7.9	6.4	7.5	6.2	7.1	8.1	8.2	6.2	6.5	7.13 (+ 0.42)
Ar	7.21	7.36	14.27	9.01	7.34	13.02	7.14	7.09	8.78	11.15 (+ 4.34)
He	0.818	0.776	0.818	0.791	0.778	0.835	0.842	0.793	0.78	0.804 (+ 0.013)

Na: Different alleles; Ne: effective alleles; Ar: allelic richness; He: expected heterozygosity; AD: Adamawa; BY: Bayelsa; EN: Enugu; OY: Oyo; IM: Imo; KN: Kano; KW: Kwara; SK: Sokoto; PL: Plateau

The distribution of Na, Ne, Ar and He per microsatellite loci across the nine States are represented in Additional files 1, 2, 3. Kruskal–Wallis test showed no significant difference between the Ar values observed across the nine States (P > 0.05). This was also observed in He and Ne values (P > 0.05).

### Parasite population differentiation

Non-random associations among loci (multilocus LD) were measured for all complete haplotypes and also those from single infections by calculating the Index of Association (IA^S^). In both the complete data set (Multi-clone and single-clone infection) and the sub-data set (single-clone infection), LD values obtained in parasite populations from Enugu, Kano, Sokoto and Plateau States were significant (P < 0.01) (Table 2). It is observed that three of the four parasite populations with significant LD were from 2018 (i.e., Enugu, Kano and Plateau) while just one (Sokoto) was from 2014. Pairwise genetic differentiation (Fst) among study sites ranged from low to moderate (Table 3). The lowest genetic differentiation was observed between Imo (South-East 1) and Kwara (North Central 1) 0.008 while the largest genetic differentiation was observed between Sokoto (North-West 1) and Oyo (South West) 0.105 (this still represents moderate genetic differentiation) (Table 3).

**Table 2:** Linkage disequilibrium analysis for *P. falciparum* populations obtained in each state

Population	*I*^*S*^_*A*_ (All)	*P-Value*	*I*^*S*^_*A*_ (Single-clone)	*P-Value*
Adamawa	0.0071	0.092	0.0031	0.375
Bayelsa	0.0118	0.042	0.0126	0.072
Enugu	0.013	< 0.001	0.0242	< 0.001
Oyo	0.0131	0.02	0.0124	0.167
Imo	0.0045	0.2	− 0.0104	0.904
Kano	0.0153	0.001	0.0149	0.002
Kwara	− 0.0070	0.924	0.0036	0.292
Sokoto	0.0167	< 0.001	0.0321	0.008
Plateau	0.0100	0.001	0.0297	< 0.001
ALL	0.0065	< 0.001	0.0073	< 0.001

*I*^*S*^_*A*_: Linkage disequilibrium

^*^Significant levels for a test of departure from 0 for *I*^*S*^_*A*_ values (P < 0.01)

**Table 3:** Pairwise comparison of Fst values amongst populations

	AD	BY	IM	EN	KW	PL	SK	KN	OY
AD	0	0.022	0.02	0.082	0.019	0.055	0.022	0.066	0.09
BY	0.022	0	0.018	0.098	0.018	0.064	0.021	0.08	0.102
IM	0.02	0.018	0	0.089	0.008	0.055	0.013	0.08	0.1
EN	0.082	0.098	0.089	0	0.093	0.055	0.099	0.024	0.051
KW	0.019	0.018	0.008	0.093	0	0.058	0.01	0.079	0.101
PL	0.055	0.064	0.055	0.055	0.058	0	0.065	0.047	0.067
SK	0.022	0.021	0.013	0.099	0.01	0.065	0	0.082	0.105
KN	0.066	0.08	0.08	0.024	0.079	0.047	0.082	0	0.042
OY	0.09	0.102	0.1	0.051	0.101	0.067	0.105	0.042	0

AD: Adamawa; BY: Bayelsa; EN: Enugu; OY: Oyo; IM: Imo; KN: Kano; KW: Kwara; SK: Sokoto; PL: Plateau

^**+**^P-value < 0.001. Fst value between 0–0.05 (little genetic differentiation), 0.05–0.15 (moderate genetic differentiation), 0.15–0.25 (great genetic differentiation), < 0.25 (very great genetic differentiation) * Interpretation is based on recommendations of Balloux and Lugon-Moulin, 2002

**Fig. 2:**
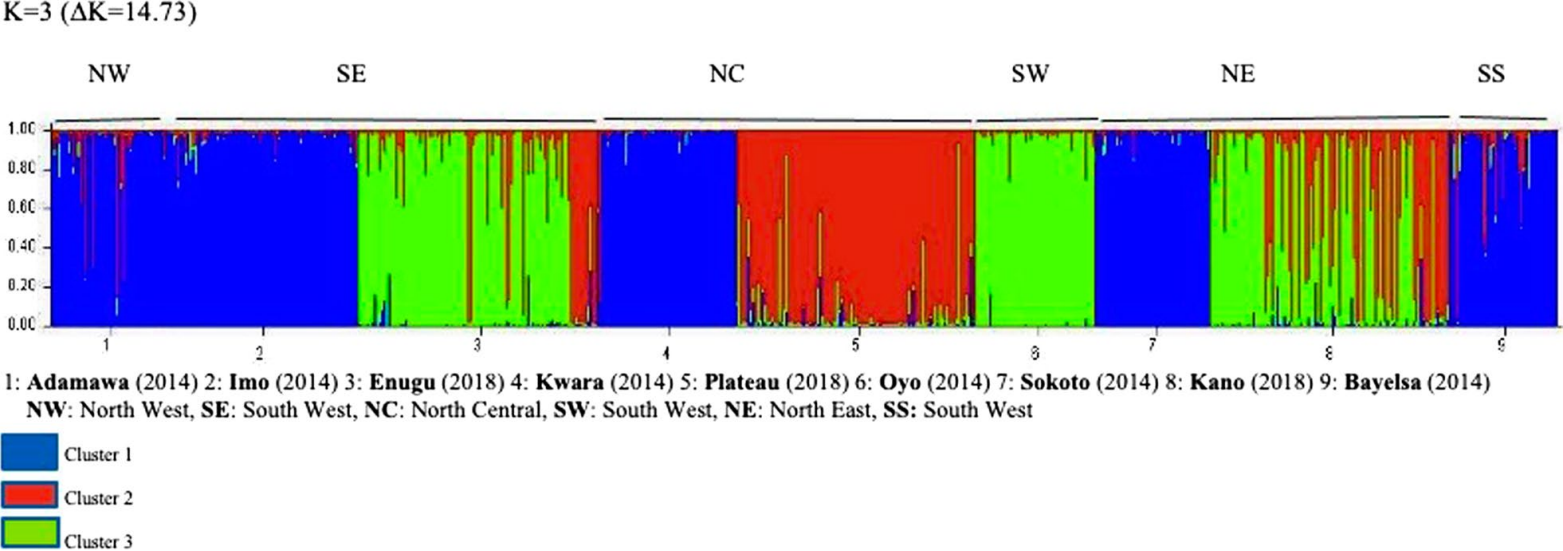
*Plasmodium falciparum* population structure in nine Nigerian States

The AMOVA result (0.039) further confirmed the low parasite genetic differentiation as just 3.9% of genetic variation were observed between study sites. Furthermore, cluster analysis using STRUCTURE confirmed low population differentiation as only three putative parasite clusters; CL 1, CL 2 and CL 3 (ΔK = 14.73) were identified as admixtures in the nine States (Fig. 2). Furthermore, the majority of parasite populations from 2014 (Adamawa, Bayelsa, Imo, Sokoto and Kwara) were in the first cluster (blue) with the exception of Oyo (Green). While in 2018, two clusters were observed (Red and Green). In Enugu, most parasites were in the third cluster (green) while in Plateau, most parasites were in the second cluster (red). Parasites from Kano State clustered almost evenly between both cluster 2 and 3.

## Discussion

Nigeria remains the country with the highest global malaria burden. Hence, molecular studies on *P. falciparum* diversity and population structure become essential in monitoring the impact of different intervention strategies in the control of malaria transmission. This study employed the use of 12 microsatellite loci to evaluate *P. falciparum* genetic diversity and population structure in nine Nigerian States. Although microsatellite are better alternatives to polymorphic markers, such as *msp*-1, *msp*-2, and *glurp*, there are only a few reports of its use in studies conducted in Nigeria [6]. Analysis of the microsatellite data generated in this study revealed high parasite genetic diversity across all States. For instance, it was observed that the mean Ne (computed as the average of the sum of Ne values across the 12 microsatellite loci) in the nine States, ranged from 6.2–8.2. This is expected because the number of Ne detected per locus is likely to be high in areas with high malaria endemicity and vice versa [5, 19].

As such, this study’s Ne values were comparable to those reported in other high-endemic regions of Sub-Saharan Africa [4, 5, 38]. Similarly, values of allelic richness (Ar) obtained in this study further confirmed the high level of parasite genetic diversity in all nine States (range: 7.09–14.27) and identical to those reported in other malaria endemic countries [39]. Furthermore, computed expected heterozygosity (He) values observed in all nine States were high ranging from 0.776–0.842. This is similar to those reported in other malaria endemic countries [5, 6, 18, 40, 41]. This further emphasizes the earlier conclusion from this study, high parasite genetic diversity and parasite transmission within the country [42]. It is equally important to note that when samples were stratified as Northern States (Adamawa, Kano, Kwara, Plateau and Sokoto) and Southern States (Bayelsa, Enugu, Imo, and Oyo) in a bid to investigate the possible influence of geographic location of observed parasite diversity, there was no significant difference in measures of genetic diversity i.e., Ne, He, and Ar values (P > 0.05). This is equally expected as malaria endemicity continues to be high across the country.

Although parasite genetic diversity was high, further analysis of microsatellite data revealed low parasite population differentiation. It was observed that when all nine States were considered as a single population, the overall association index was 0.0065 (P < 0.01), which is weaker than those typically reported in regions with low transmission [21, 23]. Studies have associated low LD values such as those reported in this study, to high levels of malaria transmission; which leads to increased cross-breeding and meiotic recombination that results in LD breakdown [5, 6, 19, 43]. The pairwise genetic differentiation (Fst) among study sites showed low to moderate genetic variation (0.008–0.105; P < 0.001). This is similar to what was reported earlier in Nigeria [6]. Furthermore, the observed low population differentiation was confirmed by AMOVA (0.039). This implies that only about 3.9% of genetic differentiation exist amongst the nine States investigated. Cluster analysis also showed that only three parasite clusters exist amongst all the nine States. However, the majority of parasite populations from 2014 (Adamawa, Bayelsa, Imo, Sokoto and Kwara) were in the first cluster (blue) with the exception of Oyo (Green). While in 2018, parasites from Enugu were in the third cluster (green), those from Plateau were in the second cluster (red) and those from Kano were distributed between cluster 2 and 3. Although, samples analysed in this work were representative of the country as a whole, a major limitation was that samples from each State were only collected at a single time point (i.e., either 2014 or 2018) thus, a spatio-temporal analysis could not be done. This perhaps would have provided more insights into the variations in clustering patterns observed in this study.

In summary, it has been observed that parasites from areas of low malaria transmission [19] (< 1% infection) show less genetic diversity, more population structure and greater linkage disequilibrium [4, 19–21]. In this study, the contrary has been observed i.e., high genetic diversity, low population structure and weak linkage disequilibrium. This is typical in regions of high malaria transmission, as individuals are more likely to be infected by more than one *P*. *falciparum* parasite thereby resulting in an increase in the rate of recombination and subsequently, high diverse population with low linkage disequilibrium [18, 19, 22]. It is plausible that the low to moderate genetic differentiation between States observed is as a result of immense human migration between these populations as part of the usual socioeconomic activities and indiscriminate vector migration within the country [6, 37, 44, 45].

## Conclusion

This study represents the first use of 12 microsatellite loci to characterize parasite genetic diversity and structure in Nigeria across regions representing all the six geographical zones of the country. The high level of parasite genetic diversity and low population structuring in this study suggests that parasite transmission is high and circulating parasites may be homogenous. However, higher resolution methods such as the 24 SNP barcode and whole genome sequencing may capture more specific parasite genetic signatures circulating in the country. The results obtained in this study can be used as a baseline for parasite genetic diversity and structure, aiding in the formulation of appropriate therapeutic and control strategies in Nigeria.

## Supplementary Information

**Additional file 1. **Shows the Number of different and effective alleles in parasite populations according to study location.

**Additional file 2. **Shows the allelic richness values in parasite populations per microsatellite loci.

**Additional file 3.** Shows expected heterozygosity (He) values of microsatellite loci from parasite populations in the nine States.

## Data Availability

The data analysed for this manuscript is available upon request from the corresponding author.

## Abbreviations

ACT: Artemisinin-based combination therapy
AMOVA: Analysis of molecular variance
Ar: Allelic richness
DBS: Dried blood spot
FST: Fixation index
GLURP: Glutamate-rich protein
He: Expected heterozygosity
LD: Linkage disequilibrium
LLIN: Long lasting insecticide net
MSP1: Merozoite surface protein 1
MSP2: Merozoite surface protein 2
Na: Number of alleles
Ne: Number of effective alleles
PCR: Polymerase chain reaction

## References

[1] Ceesay SJ, Casals-Pascual C, Erskine J, Anya SE, Duah NO, Fulford AJC (2008). Changes in malaria indices between 1999 and 2007 in The Gambia: a retrospective analysis. Lancet.

[2] O’Meara WP, Bejon P, Mwangi TW, Okiro EA, Peshu N, Snow RW (2008). Effect of a fall in malaria transmission on morbidity and mortality in Kilifi. Kenya Lancet.

[3] Jaenisch T, Sullivan DJ, Dutta A, Deb S, Ramsan M, Othman MK (2010). Malaria incidence and prevalence on Pemba island before the onset of the successful control intervention on the Zanzibar archipelago. Malar J.

[4] Bogreau H, Renaud F, Bouchiba H, Durand P, Assi S-B, Henry M-C (2006). Genetic diversity and structure of African *Plasmodium falciparum* populations in urban and rural areas. Am J Trop Med Hyg.

[5] Mulenge FM, Hunja CW, Magiri E, Culleton R, Kaneko A, Aman RA (2016). Genetic diversity and population structure of *Plasmodium falciparum* in Lake Victoria Islands, a region of intense transmission. Am J Trop Med Hyg.

[6] Oyebola MK, Idowu ET, Olukosi YA, Iwalokun BA, Agomo CO, Ajibaye OO (2014). Genetic diversity and complexity of *Plasmodium falciparum* infections in Lagos Nigeria. Asian Pac J Trop Biomed.

[7] Ariey F, Chalvet W, Hommel D, Peneau C, Hulin A, Mercereau-Puijalon O (1999). *Plasmodium falciparum* parasites in French Guiana: limited genetic diversity and high selfing rate. Am J Trop Med Hyg.

[8] Haddad D, Snounou G, Mattei D, Enamorado IG, Figueroa J, Ståhl S (1999). Limited genetic diversity of *Plasmodium falciparum* in field isolates from Honduras. Am J Trop Med Hyg.

[9] Atroosh WM, Al-Mekhlafi HM, Mahdy MA, Saif-Ali R, Al-Mekhlafi AM, Surin J (2011). Genetic diversity of *Plasmodium falciparum* isolates from Pahang, Malaysia based on MSP-1 and MSP-2 genes. Parasit Vectors.

[10] Engelbrecht F, Tögel E, Beck HP, Enwezor F, Oettli A, Felger I (2000). Analysis of *Plasmodium falciparum* infections in a village community in Northern Nigeria: determination of msp2 genotypes and parasite-specific IgG responses. Acta Trop.

[11] Happi CT, Gbotosho GO, Sowunmi A, Falade CO, Akinboye DO, Gerena L (2004). Molecular analysis of *Plasmodium falciparum* recrudescent malaria infections in children treated with chloroquine in Nigeria. Am J Trop Med Hyg.

[12] Amodu OK, Adeyemo AA, Ayoola OO, Gbadegesin RA, Orimadegun AE, Akinsola AK (2005). Genetic diversity of the msp-1 locus and symptomatic malaria in south-west Nigeria. Acta Trop.

[13] Olasehinde GI, Yah CS, Singh R, Ojuronbge OO, Ajayi AA, Valecha N (2012). Genetic diversity of *Plasmodium falciparum* field isolates from south western Nigeria. Afr Health Sci.

[14] Oyedeji SI, Awobode HO, Kun J (2013). Limited genetic diversity and low multiplicity of *Plasmodium falciparum* infections in children with severe malaria in Lafia North-central Nigeria. J Exp Clin Med.

[15] Hughes AL (1992). Positive selection and interallelic recombination at the merozoite surface antigen-1 (MSA-1) locus of *Plasmodium falciparum*. Mol Biol Evol.

[16] Hughes MK, Hughes AL (1995). Natural selection on *Plasmodium* surface proteins. Mol Biochem Parasitol.

[17] Escalante AA, Lal AA, Ayala FJ (1998). Genetic polymorphism and natural selection in the malaria parasite *Plasmodium falciparum*. Genetics.

[18] Mobegi VA, Loua KM, Ahouidi AD, Satoguina J, Nwakanma DC, Amambua-Ngwa A (2012). Population genetic structure of *Plasmodium falciparum* across a region of diverse endemicity in West Africa. Malar J.

[19] Anderson TJ, Haubold B, Williams JT, Estrada-Franco JG, Richardson L, Mollinedo R (2000). Microsatellite markers reveal a spectrum of population structures in the malaria parasite *Plasmodium falciparum*. Mol Biol Evol.

[20] Bonizzoni M, Afrane Y, Baliraine FN, Amenya DA, Githeko AK, Yan G (2009). Genetic structure of *Plasmodium falciparum* populations between lowland and highland sites and antimalarial drug resistance in Western Kenya. Infect Genet Evol.

[21] Larrañaga N, Mejía RE, Hormaza JI, Montoya A, Soto A, Fontecha GA (2013). Genetic structure of *Plasmodium falciparum* populations across the Honduras-Nicaragua border. Malar J.

[22] Volkman SK, Neafsey DE, Schaffner SF, Park DJ, Wirth DF (2012). Harnessing genomics and genome biology to understand malaria biology. Nat Rev Genet.

[23] Pumpaibool T, Arnathau C, Durand P, Kanchanakhan N, Siripoon N, Suegorn A (2009). Genetic diversity and population structure of *Plasmodium falciparum* in Thailand, a low transmission country. Malar J.

[24] Branch OH, Sutton PL, Barnes C, Castro JC, Hussin J, Awadalla P (2011). *Plasmodium falciparum* genetic diversity maintained and amplified over 5 years of a low transmission endemic in the Peruvian Amazon. Mol Biol Evol.

[25] Nkhoma SC, Nair S, Al-Saai S, Ashley E, McGready R, Phyo AP (2013). Population genetic correlates of declining transmission in a human pathogen. Mol Ecol.

[26] Bankole BE, Kayode AT, Nosamiefan IO, Eromon P, Baniecki ML, Daniels RF (2018). Characterization of *Plasmodium falciparum* structure in Nigeria with malaria SNPs barcode. Malar J.

[27] Anderson TJ, Su XZ, Bockarie M, Lagog M, Day KP (1999). Twelve microsatellite markers for characterization of *Plasmodium falciparum* from finger-prick blood samples. Parasitology.

[28] Peakall R, Smouse PE (2012). GenAlEx 6.5: genetic analysis in Excel. Population genetic software for teaching and research–an update. Bioinformatics.

[29] Excoffier L, Laval G, Schneider S (2007). Arlequin (version 3.0): an integrated software package for population genetics data analysis. Evol Bioinform Online..

[30] Ferreira MU, Nair S, Hyunh TV, Kawamoto F, Anderson TJ (2002). Microsatellite characterization of *Plasmodium falciparum* from cerebral and uncomplicated malaria patients in southern Vietnam. J Clin Microbiol.

[31] Goudet J (1995). FSTAT (Version 1.2): a computer program to calculate F-statistics. J Hered..

[32] Balloux F, Lugon-Moulin N (2002). The estimation of population differentiation with microsatellite markers. Mol Ecol.

[33] Pritchard JK, Stephens M, Donnelly P (2000). Inference of population structure using multilocus genotype data. Genetics.

[34] Falush D, Stephens M, Pritchard JK (2003). Inference of population structure using multilocus genotype data: linked loci and correlated allele frequencies. Genetics.

[35] Earl DA, von Holdt BM (2012). STRUCTURE HARVESTER: a website and program for visualizing STRUCTURE output and implementing the Evanno method. Conservation Genet Resour.

[36] Haubold B, Hudson RR (2000). LIAN 30: detecting linkage disequilibrium in multilocus data. Linkage Analysis. Bioinformatics.

[37] Schultz L, Wapling J, Mueller I, Ntsuke PO, Senn N, Nale J (2010). Multilocus haplotypes reveal variable levels of diversity and population structure of *Plasmodium falciparum* in Papua New Guinea, a region of intense perennial transmission. Malar J.

[38] Zhong D, Afrane Y, Githeko A, Yang Z, Cui L, Menge DM (2007). *Plasmodium falciparum* genetic diversity in western Kenya highlands. Am J Trop Med Hyg.

[39] Menegon M, Bardají A, Martínez-Espinosa F, Bôtto-Menezes C, Ome-Kaius M (2016). Microsatellite genotyping of *Plasmodium vivax* isolates from pregnant women in four malaria endemic countries. PLoS ONE.

[40] Conway DJ, Machado RL, Singh B, Dessert P, Mikes ZS, Povoa MM (2001). Extreme geographical fixation of variation in the *Plasmodium falciparum* gamete surface protein gene Pfs48/45 compared with microsatellite loci. Mol Biochem Parasitol.

[41] Durand P, Michalakis Y, Cestier S, Oury B, Leclerc MC, Tibayrenc M (2003). Significant linkage disequilibrium and high genetic diversity in a population of *Plasmodium falciparum* from an area (Republic of the Congo) highly endemic for malaria. Am J Trop Med Hyg.

[42] Mwingira F, Nkwengulila G, Schoepflin S, Sumari D, Beck H-P, Snounou G (2011). *Plasmodium falciparum msp1*, *msp2* and *glurp* allele frequency and diversity in sub-Saharan Africa. Malar J.

[43] Anthony TG, Conway DJ, Cox-Singh J, Matusop A, Ratnam S, Shamsul S (2005). Fragmented population structure of *Plasmodium falciparum* in a region of declining endemicity. J Infect Dis.

[44] Lum JK, Kaneko A, Tanabe K, Takahashi N, Björkman A, Kobayakawa T (2004). Malaria dispersal among islands: human mediated *Plasmodium falciparum* gene flow in Vanuatu Melanesia. Acta Trop.

[45] Lum JK, Kaneko A, Taleo G, Amos M, Reiff DM (2007). Genetic diversity and gene flow of humans, *Plasmodium falciparum*, and *Anopheles farauti s.s.* of Vanuatu: inferred malaria dispersal and implications for malaria control. Acta Trop..

