# Supplementary material for: Genetic diversity and population structure of Plasmodium falciparum in Nigeria: insights from microsatellite loci analysis
Source: Malar J. 2021 May 26;20:236. doi: 10.1186/s12936-021-03734-x (PMC8152046; doi:10.1186/s12936-021-03734-x)
Supplement: Supplementary file 1 — Additional file 1. Shows the Number of different and effective alleles in parasite populations according to study location. [file 12936_2021_3734_MOESM1_ESM.pdf]

**State**	Adamawa		Bayelsa		Enugu		Oyo		Imo		Kano		Kwara		Sokoto		Plateau	
**Locus**	Na	Ne	Na	Ne	Na	Ne	Na	Ne	Na	Ne	Na	Ne	Na	Ne	Na	Ne	Na	Ne
PolyA	26	18.9	20	12.4	35	20.4	22	12.9	24	18.4	36	20.2	27	16.3	22	14.5	23	11.7
PfPK2	14	8.0	14	7.3	16	6.7	13	8.4	10	7.4	17	8.2	12	5.8	11	6.8	10	6.0
Ta81	13	7.4	15	6.6	15	6.6	12	7.0	15	8.7	18	6.5	19	10.4	14	6.1	13	5.6
ARA2	19	15.4	20	13.8	20	10.5	17	9.8	14	9.3	22	10.5	17	12.0	10	3.8	17	10.1
TA87	23	14.6	16	8.8	19	11.8	15	9.8	17	12.1	22	9.7	19	12.9	20	11.7	19	13.8
TA40	10	6.1	13	4.5	13	4.0	10	3.4	8	2.1	19	10.1	10	4.7	16	9.1	9	2.9
TA42	10	2.2	4	1.3	11	2.7	5	2.2	7	2.3	9	2.7	9	3.2	9	3.0	7	1.5
2490	6	2.8	6	2.7	11	3.9	5	2.6	6	3.5	12	4.6	8	5.1	7	3.4	8	3.5
TA1	10	6.2	13	6.6	21	8.8	13	4.8	18	11.2	18	10.4	21	14.0	12	2.6	14	6.2
PFG377	7	3.4	9	3.0	8	3.4	5	3.5	6	3.0	6	3.2	8.	2.9	9	3.8	6	2.9
TA109	10	4.1	11	5.6	15	7.0	10	6.1	11	5.4	17	7.4	13	5.8	10	6.5	15	6.9
TA60	10	6.2	7	4.5	11	3.6	10	3.6	4	2.1	11	3.5	10	5.7	9	3.2	12	6.7
Mean	13.2	7.9	12.3	6.4	16.3	7.5	11.4	6.2	11.7	7.1	17.3	8.1	14.4	8.2	12.4	6.2	12.8	6.5
SE	1.8	1.6	1.5	1.1	2.1	1.5	1.5	0.99	1.7	1.5	2.2	1.4	1.7	1.3	1.4	1.1	1.5	1.1

Additional File 1: shows the Number of different and effective alleles in parasite populations according to study location
