# Supplementary material for: Genetic diversity and population structure of Plasmodium falciparum in Nigeria: insights from microsatellite loci analysis
Source: Malar J. 2021 May 26;20:236. doi: 10.1186/s12936-021-03734-x (PMC8152046; doi:10.1186/s12936-021-03734-x)
Supplement: Supplementary file 2 — Additional file 2. Shows the allelic richness values in parasite populations per microsatellite loci. [file 12936_2021_3734_MOESM2_ESM.pdf]

Loci	AD	BY	IM	SK	KW	KN	EN	PL	OY	Combined
PolyA	14.107	11.245	11.632	11.564	11.647	28.603	27.535	14.789	14.893	21.073
PfPK2	9.029	9.168	8.44	8.842	8.815	12.259	11.879	7.836	8.702	10.427
Ta81	7.059	8.17	7.357	6.994	7.353	14.178	17.527	9.287	11.147	12.257
ARA2	8.423	8.448	9.367	9.489	10.593	15.452	18.123	10.86	9.581	13.845
TA87	8.284	9.336	10.199	8.473	10.884	12.875	20.262	13.58	11.12	14.414
TA40	4	8.721	8.144	7.739	7.115	9	9.021	6.029	6	9.021
TA42	3.492	2.203	2.343	2.431	2.809	9.349	9.44	4.007	5.563	6.293
2490	4.73	4.544	4.481	4.892	4.04	9.076	6.904	6.379	5.874	6.822
TA1	7.621	8.321	7.117	7.01	7.441	17.37	21.477	9.418	9.461	14.308
PFG377	6.59	6.048	5.636	4.889	4.136	6.128	7.127	5.106	6.029	6.0857
TA109	6.694	6.498	7.523	6.419	5.689	13.122	12.359	9.162	9.954	10.574
TA60	6.504	5.596	5.835	6.305	5.204	8.786	9.58	8.793	9.752	8.636
Mean	7.2	7.4	7.3	7.1	7.1	13.0	14.3	8.8	9.0	11.1
SE	2.8	2.5	2.5	2.4	2.9	5.9	6.6	3.2	2.8	4.3

AD: Adamawa, BY: Bayelsa, IM: Imo, SK: Sokoto, KW: Kwara, KN: Kano, EN: Enugu, PL: Plateau and OY: Oyo

Additional File 2: shows the allelic richness values in parasite populations per microsatellite loci
