# Supplementary material for: Genetic diversity and population structure of Plasmodium falciparum in Nigeria: insights from microsatellite loci analysis
Source: Malar J. 2021 May 26;20:236. doi: 10.1186/s12936-021-03734-x (PMC8152046; doi:10.1186/s12936-021-03734-x)
Supplement: Supplementary file 3 — Additional file 3. Shows expected heterozygosity (He) values of microsatellite loci from parasite populations in the nine States. [file 12936_2021_3734_MOESM3_ESM.docx]

| Locus | Adamawa | Bayelsa | Enugu | Oyo | Imo | Kano | Kwara | Sokoto | Plateau |
| --- | --- | --- | --- | --- | --- | --- | --- | --- | --- |
| PolyA | 0.957 | 0.930 | 0.956 | 0.932 | 0.955 | 0.955 | 0.947 | 0.941 | 0.921 |
| PfPK2 | 0.884 | 0.872 | 0.856 | 0.890 | 0.873 | 0.882 | 0.834 | 0.862 | 0.841 |
| Ta81 | 0.875 | 0.859 | 0.854 | 0.866 | 0.894 | 0.851 | 0.912 | 0.844 | 0.828 |
| ARA2 | 0.945 | 0.938 | 0.909 | 0.907 | 0.902 | 0.909 | 0.925 | 0.746 | 0.908 |
| TA87 | 0.941 | 0.897 | 0.920 | 0.907 | 0.927 | 0.902 | 0.931 | 0.924 | 0.934 |
| TA40 | 0.846 | 0.786 | 0.756 | 0.714 | 0.533 | 0.906 | 0.793 | 0.899 | 0.654 |
| TA42 | 0.543 | 0.245 | 0.636 | 0.561 | 0.579 | 0.634 | 0.692 | 0.674 | 0.331 |
| 2490 | 0.649 | 0.635 | 0.747 | 0.628 | 0.723 | 0.788 | 0.809 | 0.715 | 0.721 |
| TA1 | 0.846 | 0.858 | 0.891 | 0.799 | 0.920 | 0.909 | 0.937 | 0.626 | 0.845 |
| PFG377 | 0.716 | 0.676 | 0.708 | 0.722 | 0.674 | 0.694 | 0.661 | 0.738 | 0.659 |
| TA109 | 0.765 | 0.830 | 0.862 | 0.846 | 0.822 | 0.869 | 0.835 | 0.854 | 0.861 |
| TA60 | 0.846 | 0.789 | 0.723 | 0.726 | 0.537 | 0.719 | 0.832 | 0.694 | 0.857 |
| Mean | 0.818 | 0.776 | 0.818 | 0.791 | 0.778 | 0.835 | 0.842 | 0.793 | 0.780 |
| SE | 0.037 | 0.055 | 0.029 | 0.035 | 0.046 | 0.029 | 0.027 | 0.031 | 0.049 |

**Additional File 3**: shows expected heterozygosity (He) values of microsatellite loci from parasite populations in the nine States
